# Acute insulin resistance stimulates and insulin sensitization attenuates vascular smooth muscle cell migration and proliferation

**DOI:** 10.14814/phy2.12123

**Published:** 2014-08-19

**Authors:** Eugenio Cersosimo, Xiaojing Xu, Sikarin Upala, Curtis Triplitt, Nicolas Musi

**Affiliations:** 1 Department of Medicine, Division of Diabetes and the Texas Diabetes Institute, University Health System and the University of Texas Health Science Center at San Antonio, San Antonio, Texas; 2 Department of Preventive and Social Medicine, Faculty of Medicine Siriraj Hospital, Mahidol University, Bangkok, Thailand

**Keywords:** Cell proliferation, hyperinsulinemia, inflammation, insulin signaling, migration, vascular dysfunction, vascular smooth muscle cells

## Abstract

Differential activation/deactivation of insulin signaling, PI‐3K and MAP‐K pathways by high glucose and palmitate, with/out the insulin sensitizer pioglitazone (PIO), have been previously shown in vascular smooth muscle cells (VSMCs). To determine the biological impact of these molecular changes, we examined VSMC migration and proliferation (“M”&”P”) patterns in similar conditions. VSMCs from healthy human coronary arteries were incubated in growth medium and “M”&”P” were analyzed after exposure to high glucose (25 mmol/L) ± palmitate (200 *μ*mol/L) and ± PIO (8 *μ*mol/L) for 5 h. “M”&”P” were assessed by: (1) polycarbonate membrane barrier with chemo‐attractants and extended cell protrusions quantified by optical density (OD_595_ nm); (2) % change in radius area (2D Assay) using inverted microscopy images; and (3) cell viability assay expressed as cell absorbance (ABS) in media. “M” in 25 mmol/L glucose media increased by ~25% from baseline and % change in radius area rose from ~20% to ~30%. The addition of PIO was accompanied by a significant decrease in “M” from 0.25 ± 0.02 to 0.19 ± 0.02; a comparable decline from 0.25 ± 0.02 to 0.18 ± 0.02 was also seen with 25 mmol/L of glucose +200 *μ*mol/L of palmitate. When PIO was coincubated with high glucose plus palmitate there was a 50% reduction in % change in radius. A ~10% increase in ABS, reflecting augmented “P” in media with 25 mmol/L glucose versus control was documented. The addition of PIO reduced ABS from 0.208 ± 0.03 to 0.183 ± 0.06. Both high glucose and palmitate showed ABS of ~0.140 ± 0.02, which decreased with PIO to ~0.120 ± 0.02, indicating “P” was reduced. Conclusion: These results confirm that high glucose and palmitate stimulate VSMCs migration and proliferation in vitro, which is attenuated by coincubation with the insulin sensitizer PIO. Although, we cannot ascertain whether these functional changes are coincident with the activation/deactivation of signal molecules, our findings are consistent with the theory that differential regulation of insulin signaling pathways in VSMCs in insulin‐resistant states plays an important role in inflammation, arterial wall thickening, and plaque formation during development of atherosclerosis.

## Abstract (precis)

This article describes findings on the degree of stimulation of migration and proliferation rates of VSMC derived from human coronary arteries in vitro. Exposure to high glucose and palmitate increases, whereas pioglitazone, an insulin sensitizing agent reduces migration and proliferation rates. Combined with previous demonstration that insulin signaling pathways are differentially regulated, these results suggest that insulin may be involved in vascular plaque formation in insulin‐resistant states, including type 2 diabetes

## Introduction

Cardiovascular (CV) disease is prevalent in obese individuals with hypertension and dyslipidemia (Haffner et al. 1998; Isomaa et al. 2001; American Heart Association 2014), and the risk of CV complications is magnified with the development of type 2 diabetes (Kannel et al. 1990; Turner et al. 1998). Glucose intolerance and insulin resistance are common metabolic abnormalities often found in these subjects characterized as having the “cardio‐metabolic syndrome” (Isomaa et al. 2001; DeFronzo 2010). The notion that insulin resistance itself plays a major role in the initiation and acceleration of atherosclerotic cardiovascular disease [ASCVD] was recently emphasized (Cersosimo and DeFronzo 2006). As an example, a 13‐year‐long trial (Peter Gaede and the group from the Steno 2008) implementing an aggressive treatment of “traditional” risks, but not addressing insulin resistance per se, showed only a modest 40–60% decrease in CV events. There are numerous other observations indicating that a residual CV risk, due to yet unidentified factor(s), still remains (Haffner et al. 1998; Cersosimo and DeFronzo 2006; DeFronzo 2010). This is particularly true in subjects with evidence of insulin resistance and in those who meet the criteria for the “cardio‐metabolic syndrome” (Ludmer et al. 1986; Kannel et al. 1990; Isomaa et al. 2001). Thus, the possibility that improving insulin sensitivity is required to slow the progression of ASCVD and provide additional reduction in CV event rates has not been fully tested.

Prolonged and sustained hyperinsulinemia is a hallmark of insulin‐resistant states, which coexist with vascular dysfunction and with proinflammatory and prothrombotic tendencies (Ross 1999; Cersosimo and DeFronzo 2006). In young and adult people with prediabetes and diabetes, endogenous hyperinsulinemia has been associated with impaired vascular reactivity (Wajcberg et al. 2006, 2007), early thickening of the carotid intima media layer (Mazzone et al. 2006) and microalbuminuria, reflecting capillary endothelial dysfunction (Steinberg et al. 1996). The fact that preatherosclerotic lesions are frequently associated with molecular abnormalities of insulin resistance (Ross 1999; Cersosimo and DeFronzo 2006; DeFronzo 2010) suggests that they might share common underlying mechanisms. In this regard, the progression of vascular stiffness to arterial wall thickening with inflammation evolving into plaque formation (Mazzone et al. 2006; Nissen et al. 2008) may be favored by intrinsic cellular metabolic defects. This relationship is illustrated by the demonstration that improper activation of insulin signaling molecules occurs concomitantly with vascular endothelial dysfunction (Cersosimo and DeFronzo 2006; Wajcberg et al. 2007; Gogg et al. 2009; Cersosimo et al. 2012). Moreover, inadequate vascular responses tend to be partially restored with interventions aimed at correcting impaired insulin signaling pathways in various tissues (Musi et al. 2001; Greco et al. 2002; Reyna et al. 2008), including vascular endothelial and smooth muscle cells (Blaschke et al. 2006; Goya et al. 2006; Gogg et al. 2009; Cersosimo et al. 2012).

Several factors are known to interfere with the activation process of the metabolic action of insulin at the cellular level (Kelley et al. 1992; Shulman 2000). The accumulation of lipids (Belfort et al. 2005) and proinflammatory cytokines represent two of the most prominent and potentially reversible findings associated with molecular insulin resistance (Shulman 2000). These have been confirmed in hepatocytes in culture and in skeletal muscle samples obtained in patients with type 2 diabetes (Rothman et al. 1992; Pessin and Saltiel 2000; Shulman 2000). Of interest, however, the activity of the insulin signaling cascade responsible for glucose transport and metabolism, that is, the phospho‐inositol‐3 kinase (PI‐3K) was found to be reduced, whereas the activation/inactivation cycle of the mitogenic (MAP‐K) insulin signaling molecules was preserved or augmented (Cusi et al. 2000). Similar results were subsequently reported in endothelial cells (Gogg et al. 2009) and in vascular smooth muscle cells (VSMCs) harvested from coronary arteries of healthy subjects in culture media containing high glucose, fatty acids, and insulin (Cersosimo et al. 2012). Differential responses of the insulin metabolic and mitogenic pathways were reversed in the presence of pioglitazone, a PPAR‐ *γ* agonist known to have metabolic insulin sensitizing properties (Goya et al. 2006; Cersosimo et al. 2012). Considering that inflammation and proliferation of VSMCs in the arterial wall represent important steps that directly contribute to the initiation and continuation of the atherosclerosis process (Ross 1999), we chose to examine some regulatory aspects of these cells. In this regard, the hypothesis that the mitogenic insulin signaling pathways may preferably be activated in the presence of hyperinsulinemia and promote cell migration and proliferation may be extended to VSMCs, which would lead to local inflammation and arterial wall thickening with plaque formation.

In this study, we investigated the degree of migration and proliferation of VSMCs obtained from normal human coronary arteries cultured in media mimicking the insulin‐resistant state. Cells were exposed to acute hyperinsulinemia in the presence and absence of the insulin sensitizer pioglitazone. We tested the hypotheses that under these experimental conditions, high glucose and palmitate would stimulate, while coincubation with pioglitazone would attenuate the enhanced VSMCs migration and proliferation patterns.

## Material and Methods

### Primary cell cultures

Healthy human coronary arterial smooth myoblasts (C‐12511 HCASMC‐c) were obtained from Promo Cell (Heidelberg, Germany). Cells were grown in PromoCell SMC growth medium‐2 (C‐22062 and C‐39267) that was replaced every 48 h; the cell adherence rate was checked periodically. The presence of mature and differentiated smooth muscle cells was confirmed under light microscope by the typical “spindle‐cell” appearance. Further confirmation was obtained in selected culture dishes by immunohistochemistry and by immunoelectrophoresis, after the addition of a specific *α*‐actin antibody (Sigma‐Aldrich Corp., St Louis, MO).

#### Cell viability assays

Prior to each set of experiments, cell viability was assessed (Musi et al. 2001; Cersosimo et al. 2012) and only those preparations associated with a value equal or greater than 80% cell survival were used. Cell viability testing included VSMC in media containing glucose at 5 mmol/L and 25 mmol/L and/or palmitate at 200 *μ*mol/L concentrations, all in the presence and absence of pioglitazone at the dose of 8 *μ*mol/L. Culture media concentrations of glucose of 5 mmol/L and 25 mmol/L, and of palmitate of 200 *μ*mol/L were selected because these reproduce clinically relevant circulating levels of normoglycemia, severe hyperglycemia, and elevated free‐fatty acids, respectively encountered in obese subjects and patients with type 2 diabetes (Steinberg et al. 1996; Tong et al. 2005; Goya et al. 2006; Gogg et al. 2009; DeFronzo 2010; Esfandiarei et al. 2011). Exposure of VSMC to a pioglitazone dose of 8 *μ*mol/L was utilized in all experiments in order to resemble the pharmacological levels usually attained during diabetes therapy (Wajcberg et al. 2007).

### Experimental design

#### VSMC incubation

After VSMCs differentiation and viability were ascertained, cells were seeded in triplicate in working plates (6‐well culture plates) at a density of 2 × 10^4^/well and incubated until they were ~90% confluent at 37°C in the incubator. To examine the effects of high glucose alone or in combination with palmitate, both in the absence and in the presence pioglitazone, VSMCs were treated for 24 h in culture media containing either 5 mmol/L or 25 mmol/L of glucose alone plus bovine serum albumin (BSA) and with both 25 mmol/L of glucose combined with 200 *μ*mol/L of palmitate conjugated with BSA. Similar experiments were conducted in control culture media containing 0.1% of Di‐Methyl‐Sulpha‐Oxide (DMSO) solution plus BSA. All experimental conditions were reproduced with the addition of pioglitazone, at the dose of 8 *μ*mol/L to the incubation media. At the end of the 24‐h period, insulin at the dose of 100 nmol/L was added for 20 min in all experiments and VSMCs were prepared for analyses in cell migration and proliferation assays (Cersosimo et al. 2012). Glucose was prepared with serial dilutions of a stock glucose solution prepared as 1 mol/L glucose using deionized distilled water. Stock palmitate solution was prepared with 8 mmol/L sodium palmitate conjugated with 10.5% BSA (Sigma, St Louis, MO), as previously described (Livak and Schmittgen 2001; Reyna et al. 2008). Pioglitazone was dissolved in DMSO at a final concentration of 0.1%. Control samples not treated with pioglitazone were incubated in 0.1% DMSO.

#### Migration and proliferation assays

Following exposure to the experimental conditions outlined above, the degree of VSMCs migration was evaluated by two independent methods: (1) a polycarbonate membrane barrier using chemo‐attractants with extended cell protrusions quantified by optical density (OD_595 _nm, Roche, Pleasanton, CA) and (2) assessment by inverted microscopy images (2D Assay, Cell BioLabs, San Diego, CA) with percent changes in radius area calculated using the formula:

[*π*r^2^ (BEFORE) − *π*r^2^ (AFTER)/*π*r^2^ (BEFORE)] * 100.

The polycarbonate membrane barrier cell migration assay was conducted using the CytoSelect^™^ Cell Migration Assay Kit with membrane inserts of 8 *μ*m pore size in a 24‐well plate. The membrane serves as a barrier to discriminate migratory cells from nonmigratory cells. Migratory cells are able to extend protrusions toward chemo‐attractants (via actin cytoskeleton reorganization) and ultimately pass through the pores of the polycarbonate membrane. Finally, the cells are removed from the top of the membrane and the migratory cells are stained and quantified. In this assay, VSMCs (~0.5 × 10^4^ cells/mL) in each experiment were prepared and extracted from serum‐free media to be seeded in separate wells containing 500 *μ*L of pretreatment media. After addition of 300 *μ*L of cell suspension to each insert migration plates were incubated for 24 h, in a humidified atmosphere (37°C and 5% CO_2_). Cell preparation wells were removed from the incubator and media were gently aspirated from the insert in order to remove the identified nonmigratory cells. Next, the insert was transferred to clean wells containing 400 *μ*L of cell stain solution and incubated for 10 min at room temperature. At the end, inserts were washed with water and allowed to air dry. Each insert was transferred to empty wells, 200 *μ*L of extraction solution was added, and the cell solution was incubated for 10 min on an orbital shaker. Upon completion, 100 *μ*L were removed and transferred to a 96‐well microtiter plate reader and the spectrophotometrical absorbance [optical density OD‐595 nm] was recorded. Results are expressed as percent changes induced during exposure of VSMCs to the culture media in each experimental condition and compared to values obtained with the control culture media.

In a separate assay, inverted microscopic images were obtained with the Radius Cell Migration Assay Kit utilizing a proprietary 24‐well plate to monitor the migratory properties of cells. A proprietary cell culture plate containing a carefully defined nontoxic, biocompatible hydro gel [Radius Gel spot 0.68 ± 0.014 mm in diameter] centralized at the bottom of each well was utilized. When VSMCs were seeded in the well, they attached everywhere except on the Radius Gel, creating a cell‐free zone. Following cell seeding, the Radius Gel was removed, allowing migratory cells to move across the area and close the gap. This format provides a robust in vitro system to measure 2‐D cell migration in various experimental conditions. The procedure involves placement of a culture of VSMCs under sterile conditions into each well plate pretreated with 500 *μ*L of gel solution, which were covered and incubated at room temperature for 20 min. The gel solution was then treated with 500 *μ*L of wash solution and aspirated. Cells were harvested from each individual experimental culture media and resuspended in 500 *μ*L solution (0.3 × 10^6^ cells/mL). The migration plate was transferred to a cell culture incubation media for 4–24 h, after which it was gently removed from the incubator. The aspirate was washed three times with 500 *μ*L of fresh media followed by the application of an additional 500 *μ*L of a gel removal solution, prepared by a serial dilution of the stock 1:100. The plate was again transferred to a cell culture incubator for 30 min to allow complete gel removal, and then the solution was aspirated from each well and washed three times with 500 *μ*L of fresh media.

After the final washing was completed, 1 mL of culture medium was added and each well was prepared for imaging using an inverted microscope. The plate was transferred back to the cell culture incubator/microscope stage incubator during the migration process, and closure by endpoint was monitored. VSMCs were stained with 400 *μ*L of cell stain solution added to each vial after the removal of the media from the wells. VSMCs were allowed to stain for 5–15 min at room temperature and at the end the solution was aspirated and discarded. Wells were carefully washed several times with 1 mL of deionized water and dried at room temperature. Under inverted light microscopy, the degree and patterns of VSMCs migration images were analyzed using a customized software program (CellProfiler^™^ Cell Image Analysis Software, at www.cellprofiler.org). In a series of preliminary studies, it was determined that the “best timing” to capture the migration imaging of VSMCs in culture was after approximately 5 h (Cersosimo et al. 2012). In these experiments, inspections were conducted hourly and very little to no migration was detected from 1 to 4 h of incubation. In contrast, after 6 h, VSMC migration was excessive in most conditions and the “circle” was entirely filled with cells, making it impossible to quantify cell migration with accuracy. Results are expressed as percent changes in the radius in the open gel circle determined for groups of VSMCs harvested from each experimental condition, as calculated by the formula outlined above.

VSMC proliferation was analyzed in similar experimental conditions using a Colorimetric Assay (MTT based) for nonradioactive quantification of cell proliferation and viability, as previously reported (Cersosimo et al. 2012). The spectrophotometrical absorbance of samples was measured at wavelength between 550 nm and 690 nm [A_550 _nm − A_690 _nm] using a microplate reader (Roche, Pleasanton, CA). Results were expressed as the Cell Absorbance detected in each set of experiments.

#### Statistical analyses

The main objective of these studies was to evaluate the degree of VSMCs migration and proliferation in experimental conditions comparable to those (Cersosimo et al. 2012) shown to be accompanied by changes in insulin signal transduction (Akt & MAP‐K pathways). In each experimental condition, VSMCs migration and proliferation were assessed in duplicate using two independent techniques, the Optical Density and 2D Assay (migration) and the Cell Viability assay (proliferation), as described above. Each experiment was repeated in six separate occasions (*n* = 6).

The final number of experiments needed (*n*) was calculated using a two‐tailed test, based on published data from the literature indicating a variance in the measurement of cell migration and proliferation between 4.0 and 8.0% (White et al. 2006). Statistical analysis (SigmaStat, SigmaPlot System, San Jose, CA) of continuous variables in all experimental conditions was performed using the Kruskal–Wallis rank test and the subanalysis was conducted with the Wilcoxon rank‐sum test. A *P < *0.05 was considered significant. Data are expressed as means ± SEM

## Results

**Figure 1.**
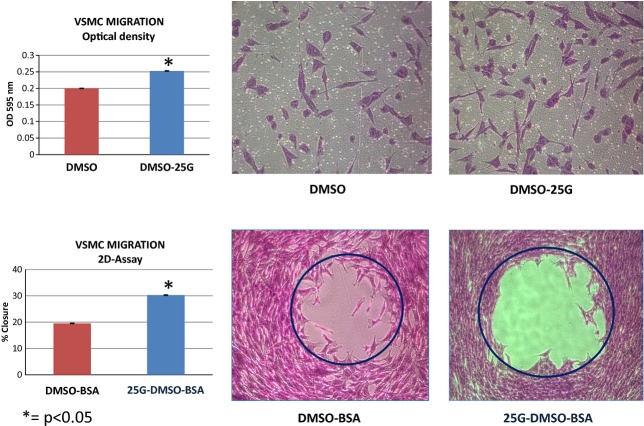
Micrographic demonstration of the effects of high‐glucose incubation followed by insulin perfusion on vascular smooth muscle cell migration documented using two independent assays: (1) Optical Density (bar graph & micrograph ON TOP) and (2) 2D‐assay (bar graph & micrograph AT BOTTOM). Exposure to 25 mmol/L glucose increased cell migration by 25%, from 0.20 ± 0.02 to 0.25 ± 0.02 (OD_595 _nm). The percent chamber area closure rose from ~20 to ~30% (2D‐assay). Percent Change Area = [*π*r^2^ (BEFORE) − *π*r^2^ (AFTER)/*π*r^2^ (BEFORE)] * 100. The experiments were performed in duplicate (*n* = 6). Data are expressed as means ± SEM. DMSO = control media; BSA = bovine serum albumin; 25G = 25 mmol/L glucose. *P < 0.05.

**Figure 2.**
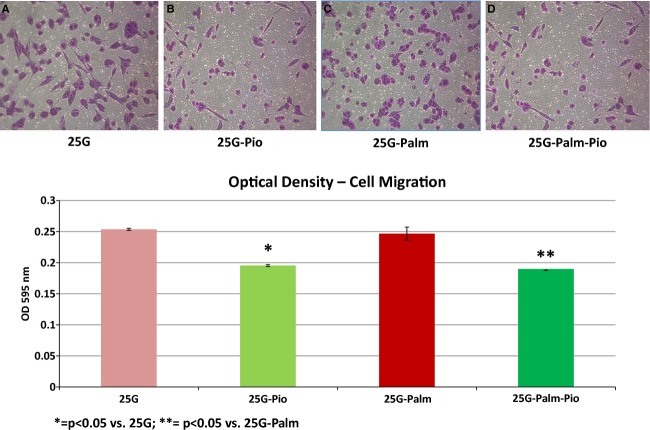
Micrographic demonstration of the effects of high glucose and palmitate incubation followed by insulin perfusion on vascular smooth muscle cell migration documented using the Optical Density Cell Migration Assay. (A) = In the presence of 25 mmol/L glucose, there was an increase in cell migration up to 0.25 ± 0.02 (OD_595_ nm), which was significantly attenuated (0.18 ± 0.02) when the insulin sensitizer pioglitazone (8 *μ*mol/L) was added to the media (B). A similar increase up to 0.24 ± 0.02 (OD_595_ nm) in cell migration was observed when the media was exposed to the combination of high glucose and 200 *μ*mol/L of palmitate (C), which was significantly reduced to 0.18 ± 0.02 with the addition of pioglitazone (D). The experiments were performed in duplicate (*n* = 6). Data are expressed as means ± SEM. 25G = 25 mmol/L glucose; Palm = 200 *μ*mol/L of palmitate; Pio = pioglitazone (8 *μ*mol/L). *P < 0.05 versus 25G; **P < 0.05 versus 25G‐Palm.

**Figure 3.**
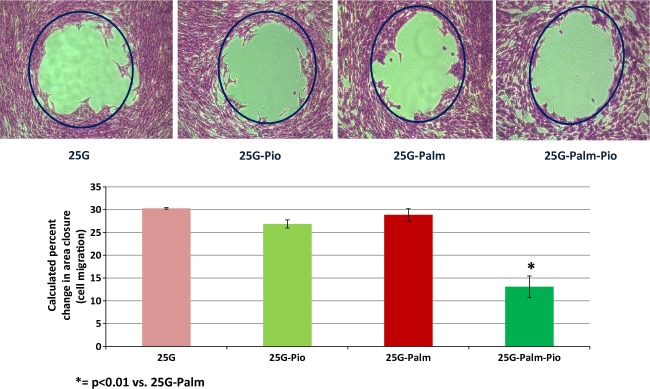
Micrographic demonstration of the migration patterns of VSMCs using the percent chamber area closure method. Graph bars show that exposure to 25 mmol/L Glucose increased the mean percent chamber area closure to ~30% (2D‐assay‐5 h); pioglitazone (Pio) addition restored the mean percent chamber area closure to ~25%. In culture media containing the combination of palmitate (200 *μ*mol/L) plus Glucose (25 mmol/L), there was an increase in the mean percent area closure to 28%; Pio addition decreased the mean percent area closure to 15%. Percent Change Area = [*π*r^2^ (BEFORE)‐*π*r^2^ (AFTER)/*π*r^2^ (BEFORE)] *100. The experiments were performed in duplicate (*n* = 6). Data are expressed as means ± SEM. 25G = 25 mmol/L glucose; Palm = 200 *μ*mol/L of palmitate; Pio = pioglitazone (8 *μ*mol/L). *P < 0.01 versus 25G‐Palm.

VSMCs in culture media containing 25 mmol/L of glucose demonstrated a ~25% increase from a baseline value of 0.20 ± 0.02 to 0.25 ± 0.02 (OD_595_ nm) in cell migration, whereas those in control media with DMSO and without glucose remained the same. A nearly 50% increase was also seen in the percent chamber area closure of ~20% at baseline to ~30% (2D assay‐5 h) in VSMCs exposed to 25 mmol/L of glucose, in contrast to those in control media with no significant change detected. Microscopic views highlighting these changes in one representative set of experiments are included (Fig. 1). When VSMCs were cultured in media with 25 mmol/L of glucose containing 8 *μ*mol/L pioglitazone there was a significant decrease in cell migration from 0.25 ± 0.02 to 0.19 ± 0.02 (OD_595 _nm). An equivalent decrease from 0.25 ± 0.02 to 0.18 ± 0.02 (OD_595 _nm) was also documented in VSMCs simultaneously exposed to 25 mmol/L of glucose plus 200 *μ*mol/L of palmitate when pioglitazone was added. One example of these changes as seen under microscopy is enclosed (Fig. 2). As shown in Fig. 3, a 50% reduction from ~28% down to ~14% in the percent chamber area closure was observed in VSMCs cultured in media with both high glucose plus palmitate in the presence of the insulin sensitizer pioglitazone. A mild nonsignificant decline from ~30% to ~26% was also registered when pioglitazone was added to VSMCs cultured in 25 mmol/L of glucose alone. These changes can be appreciated in the enclosed microscopic pictures obtained in one representative set of experiments (Fig. 3).

**Figure 4.**
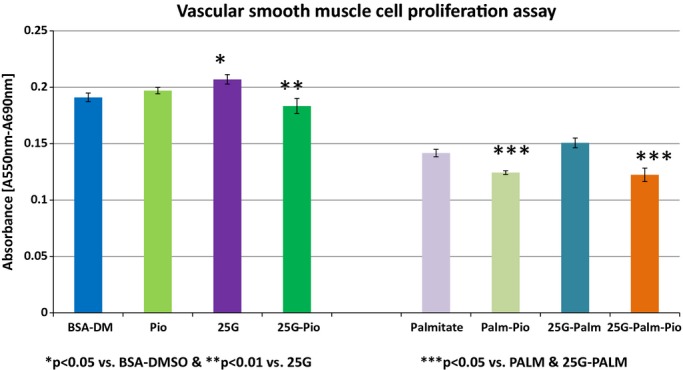
The effects of high glucose and palmitate in the absence and presence of pioglitazone on VSMCs proliferation, as determined by cell viability and expressed as ABSORBANCE [A550 nm‐A690 nm] in an assay using 3‐[4, 5‐dimethylthiazol‐2yl]‐2,5‐diphenyltetrazoliun bromide. Cell proliferation was recorded after 24 h incubation in culture media containing BSA‐DMSO plus insulin (control); pioglitazone (8 *μ*mol/L) [PIO]; 25 mmol/L glucose [25G]; r 25G plus pioglitazone [25G‐PIO]; palmitate (200 *μ*mol/L); palmitate plus pioglitazone (8 *μ*mol/L) [PALM‐PIO]; 25 mmol/L glucose plus palmitate [25G‐PALM] and 25G‐PALM plus pioglitazone [25G‐PALM‐PIO]. *P < 0.05 versus BSA‐DMSO & **P < 0.01 versus 25G; ***p<0.05 versus PALM & 25G‐PALM.

The degree of VSMCs proliferation was examined in a series of experimental conditions equal to those described above and, the results are summarized in Fig. 4. Exposure of cells to control media containing BSA and DMSO showed an absorbance of 0.190 ± 0.05 and with pioglitazone alone it was 0.197 ± 0.03. VSMCs proliferation increased and a rise in cell absorbance to 0.208 ± 0.03 when 25 mmol/L of glucose was added to the culture media was documented. A significant reduction down to 0.183 ± 0.06 was noted in cells exposed to pioglitazone. VSMCs in media containing palmitate showed a cell absorbance of 0.140 ± 0.02, which decreased to 0.121 ± 0.02 with pioglitazone. Similarly, there was an increase in cell absorbance with coincubation of 25 mmol/L of glucose plus palmitate, which also declined to 0.120 ± 0 with pioglitazone.

## Discussion

These studies demonstrate that in vitro migration and proliferation of vascular smooth muscle cells harvested from human coronary arteries are stimulated in media containing high glucose and palmitate. The increased cell migration and proliferation are substantially attenuated in the presence of the PPAR‐*γ* agonist and insulin sensitizer pioglitazone. These results provide further evidence that in conditions mimicking acute insulin resistance the enhanced migratory and proliferative response of smooth muscle cells can be mitigated by an agent known to facilitate the metabolic intracellular insulin signaling cascade. Our data are consistent with the notion that differential activation of insulin signals, that is, metabolic versus mitogenic pathways, by elevated insulin levels may be partially responsible, at least in part, for the involvement of smooth muscle cells in the process of inflammation, intima media thickening, and development of arterial plaques. Based on these findings and previous observations (Hsueh 1999; Touyz and Schiffrin 2006; Davidson et al. 2008; Draznin 2010; Esfandiarei et al. 2011), we speculate that by restoring the activity of metabolic insulin pathways [PI‐3 kinase], the alternate mitogenic MAP‐kinase pathway may be alleviated, which lessens the proliferative and inflammatory vascular response.

The importance of VSMCs migration and proliferation in the initial formation of arterial plaques and also in advanced stages in the process of atherosclerosis has been well documented (Ross 1999). There are numerous reports indicating that cellular and molecular defects in the insulin signaling pathways are present in VSMCs derived from animal models (Hsueh 1999; Blaschke et al. 2006; Esfandiarei et al. 2011), diabetic patients (White et al. 2006; Draznin 2010), and from healthy individuals in culture conditions mimicking insulin resistance (Cersosimo et al. 2012). The extent to which the mitogenic effects of insulin are responsible for the proliferation and migration of VSMCs in insulin‐resistant states, however, remains controversial (Draznin 2010). In this study, using two independent methods we have demonstrated that VSMCs cultured in media with 25 mmol/L glucose migrated at a rate of 25–30% greater than those in control media. Comparable migratory rates were documented when palmitate was added to the media containing 25 mmol/L glucose. Although we did not investigate the mechanism underlying these changes, the fact that the combination high glucose and palmitate in the culture media elicited migration rates comparable to those seen when VSMCs were exposed to high glucose alone suggests, but does not prove that a common mechanism may be involved. Cell proliferation also increased by nearly 20% in the presence of high glucose, and cell viability declined only minimally by exposure to palmitate, comparable to our previous observations (Cersosimo et al. 2012). In contrast, however, treatment with pioglitazone was associated with a consistent decline of approximately 30% in the migratory rates of cells cultured in media with either 25 mmol/L glucose and/or 200 *μ*mol/L of palmitate. In a series of experiments utilizing the percent area closure as indicator, the addition of pioglitazone to the culture media containing high glucose plus palmitate, was accompanied by a remarkable ~50% reduction in the degree of cell migration. These observations raise the possibility that palmitate mitigates the suppressing effect of pioglitazone. Alternatively, however, it is plausible that the mechanism(s) by which palmitate increases VSMCs migration overwhelms that of high glucose in the culture media and, as a consequence cells may become more sensitive to the action of pioglitazone. Since this study was not designed to specifically address the mechanisms behind these changes, these represent mere speculations. Although not quite as robust, VSMCs proliferation was also attenuated by ~15% with pioglitazone treatment in similar experimental conditions. Collectively, these observations are in agreement with the theory that high glucose and palmitate in the presence of elevated insulin levels, characteristically seen in individuals with insulin resistance with or without type 2 diabetes may be potent stimulators of VSMCs migration, proliferation, and inflammation.

We have previously shown (Cersosimo et al. 2012) that palmitate reduces the activity of the insulin‐mediated metabolic PI‐3 kinase pathway, while activating the mitogenic transduction signal in vascular smooth muscle cells. Others have noted similar effects of high glucose in the process of activation/deactivation of insulin signaling pathways in vascular endothelial cells (Gogg et al. 2009) and in skeletal muscle fibers obtained in obese diabetic patients (Cusi et al. 2000). Considering that handling and preservation of VSMCs exposed to various experimental conditions in our laboratory behave similarly to those reported in the literature (Musi et al. 2001; White et al. 2006; Draznin 2010), it is likely that the presumed changes in the activity of insulin signaling pathways (not shown in these experiments) may be the underlying molecular mechanism behind the stimulation of VSMCs migration and proliferation. It is noteworthy that enhanced smooth muscle cell migration and proliferation together with the vascular inflammatory response are recognized factors contributing to intima media thickening and arterial plaque formation, both preatherosclerotic lesions (Ross 1999; Hsueh and Law 2001; Touyz and Schiffrin 2006). The presumed association between the enhanced migratory and proliferative patterns shown here and the underlying changes in the activity of insulin molecular pathways are entirely compatible with in vitro findings (3,5,10,15,) and clinical observational data (Steinberg et al. 1996; Mazzone et al. 2006; Touyz and Schiffrin 2006; Wajcberg et al. 2007; Davidson et al. 2008; Nissen et al. 2008). These studies present convincing evidence that whereas lipids and its derivatives inhibit, thiazolidinediones tend to improve insulin–mediated glucose metabolism and correct vascular dysfunction. Although a linkage between these effects at the cellular and molecular levels remains to be determined. It must be emphasized that our results are limited to in vitro cell culture experimental conditions and thus, clinical relevance can only be implied at this stage. To advance our understanding of the molecular mechanisms underlying the accelerated process of atherosclerosis in individuals with insulin resistance, including those with type 2 diabetes, additional studies are required.

The findings that the addition of the PPAR‐*γ* agonist and insulin sensitizer pioglitazone to the glucose‐ and palmitate–rich culture media is able to attenuate the degree of migration and proliferation of the VSMCs are of considerable significance. These observations are in line with our own earlier publication and those of other investigators (Hsueh and Law 2001; Tong et al. 2005; Touyz and Schiffrin 2006; Cersosimo et al. 2012) demonstrating that while the mitogenic signaling cascade is deactivated, the insulin‐mediated metabolic pathways are stimulated in the presence of pioglitazone. One possibility is that PPAR‐*γ* agonists bind to a specific ligand and promote cell cycle arrest, via inhibition of protein phosphorylation (Hsueh 1999). Additionally, some reports have provided evidence indirectly linking the effects of PPAR‐*γ* agonists on VSMCs insulin signaling pathways to “eNOS” activation, to antifibrotic effects or even changes in intermediary signals in both vascular endothelial and inflammatory cells (Tong et al. 2005; Goya et al. 2006; Touyz and Schiffrin 2006; Pan et al. 2009). Nonetheless, the likelihood, however, that the directional changes in the activation/deactivation process of these molecular pathways serve as stimuli to the augmented VSMCs migratory and proliferative patterns should not be underestimated. Again, even though our studies are limited to in vitro cell preparations, numerous animal and clinical observations in patients with type diabetes have reported clear beneficial antiatherosclerotic effects of the PPAR‐*γ* agonist pioglitazone (Hsueh and Law 2001; Mazzone et al. 2006; Nissen et al. 2008). Therefore, we can only speculate as to the true extent of the involvement of insulin‐mediated molecular mechanisms and how critical a role they represent in the accelerated process of atherosclerosis in individuals with insulin resistance and type 2 diabetes.

In conclusion, following a line of investigation of some potential molecular mechanisms responsible for the severe and rapid progression of atherosclerosis in type 2 diabetes, our current studies confirm that in the presence of high glucose and palmitate migration and proliferation of VSMCs are stimulated. Coincubation with the insulin sensitizer pioglitazone reduces the enhanced migratory and proliferative rates of cells exposed to media mimicking insulin‐resistant conditions. Although likely, we cannot ascertain that these changes were coincident with simultaneous changes in the activation/deactivation of insulin–mediated metabolic and mitogenic pathways. Additional studies in whole animals and in humans are needed to further advance the theory that differential insulin signaling represents one important factor underlying the involvement of smooth muscle cells in atheroma plaque formation during the development of atherosclerosis.

## Conflict of Interest

None declared.
